# Autopsy Proven Pulmonary Mucormycosis Due to *Rhizopus microsporus* in a Critically Ill COVID-19 Patient with Underlying Hematological Malignancy

**DOI:** 10.3390/jof7020088

**Published:** 2021-01-27

**Authors:** Christoph Zurl, Martin Hoenigl, Eduard Schulz, Stefan Hatzl, Gregor Gorkiewicz, Robert Krause, Philipp Eller, Juergen Prattes

**Affiliations:** 1 Section of Infectious Diseases and Tropical Medicine, Department of Internal Medicine, Medical University of Graz, 8036 Graz, Austria; christoph.zurl@medunigraz.at (C.Z.); martin.hoenigl@medunigraz.at (M.H.); robert.krause@medunigraz.at (R.K.); 2 Division of General Pediatrics, Department of Pediatrics and Adolescent Medicine, Medical University of Graz, 8036 Graz, Austria; 3 BioTechMed-Graz, 8036 Graz, Austria; gregor.gorkiewicz@medunigraz.at; 4 Division of Infectious Diseases and Global Public Health, Department of Medicine, University of California San Diego, San Diego, CA 92103, USA; 5 Division of Hematology, Department of Internal Medicine, Medical University of Graz, 8036 Graz, Austria; eduard.schulz@medunigraz.at (E.S.); stefan.hatzl@medunigraz.at (S.H.); 6 Diagnostic and Research Institute of Pathology, Medical University of Graz, 8010 Graz, Austria; 7 Department of Internal Medicine, Intensive Care Unit, Medical University of Graz, 8036 Graz, Austria; philipp.eller@medunigraz.at

**Keywords:** COVID-19, mucormycosis, fungal infections, co-infections

## Abstract

Viral infections can cause acute respiratory distress syndrome (ARDS), consequently leading to susceptibility for secondary pulmonary infections. Over the past few weeks, a number of studies have reported on secondary pulmonary aspergillosis complicating severe COVID-19. We report the case of a 53-year old male patient with secondary acute myeloid leukemia (AML) who suffered from COVID-19 ARDS and was diagnosed postmortem with mucormycosis.

## 1. Introduction

Severe acute respiratory syndrome coronavirus 2 (SARS-CoV-2) is continuing to spread worldwide with a high proportion of infected individuals needing respiratory support and ICU treatment [1]. Viral infections can cause acute respiratory distress syndrome (ARDS), consequently leading to susceptibility for secondary pulmonary infections. Over the past few weeks, a number of studies have reported on secondary pulmonary aspergillosis complicating severe COVID-19 [2]. Here, we report the first case of a critically ill COVID-19 patient who was diagnosed with pulmonary mucormycosis. 

## 2. Case Report

**Figure 1:**
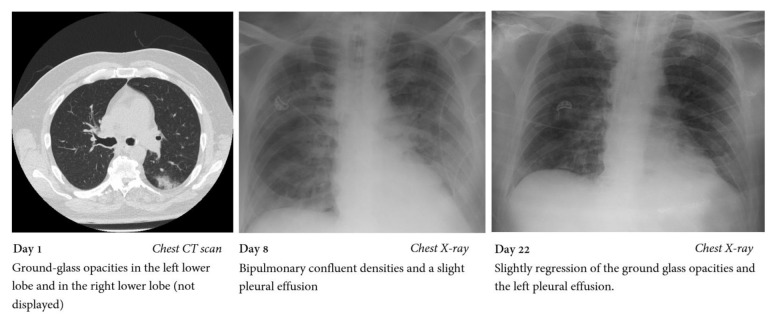
Chest CT scan and chest X-rays performed during hospital stay.

A 53-year old male patient was diagnosed with secondary acute myeloid leukemia (AML) in January 2020 and was transferred to our hospital for further treatment. Medical history included myelodysplastic syndrome, obesity (body mass index 34) and depression. Five weeks after induction with a classical “7 + 3” regimen (consisting of a seven-day treatment course with cytarabine and three days of daunorubicin) the patient developed sore throat, parageusia, dysosmia and fever up to 39 °C. Antifungal prophylaxis at this time consisted of iv voriconazole with 400 mg twice daily on day 1, followed by 200 mg twice daily thereafter (therapeutic drug-monitoring adapted, with a trough voriconazole plasma level of 3.37 mg/L). While breathing ambient air, oxygen saturation was 96% and chest CT-scan showed slight bilateral infiltrates (Figure 1). Polymerase chain reaction (PCR) for SARS-CoV-2 from a nasopharyngeal swab was performed and revealed a positive result 54 days after induction chemotherapy. Due to development of moderate acute respiratory distress syndrome (oxygenation index (OI) 115)) the patient was admitted to the intensive care unit for noninvasive ventilation five days after being tested positive. At the time of ICU admission, the patient had suffered from severe neutropenia (<500 neutrophils/µL) for eight weeks and presented with a neutrophil count of 300/µL, platelet count of 8000/µL and lymphocyte count of 100/µL. Therapy for COVID-19 ARDS at this stage of the pandemic consisted of tocilizumab and high-dose glucocorticoids (starting with prednisolone 100 mg for two days followed by continuously tapering with total duration of 17 days until death). Chest X-ray showed an increase of bilateral infiltrates and the patient developed severe ARDS (OI 60). Thus, he was intubated on day 8 after symptom onset (day 3 in the ICU), and bronchoscopy and bronchoalveolar lavage (BAL) was performed. BAL galactomannan (GM) and culture as well as serial serum GM testings remained negative. Serum 1,3-ß-D-glucan (BDG) was positive only once (178 pg/mL) during ICU stay. Neutrophils increased over 500/µL from day 16 onwards. After a switch of antibiotic therapy for piperacillin/tazobactam plus linezolid, the patient improved slightly and was extubated at day 18. Shortly after, however, he developed fever up to 39.5 °C. Blood culture, Acridine-Orange Leucocyte Cytospin test, viral PCRs and fungal biomarkers were negative. Due to rapid respiratory deterioration on day 22 the patient was reintubated. SARS-CoV-2 PCR from nasopharyngeal swab was performed and was still positive, and culture from BAL showed mixed nonpathogenic flora. Chest X-ray did not show any new infiltrates. Hemodynamic situation worsened within the next days, and the patient died on day 24 after symptoms onset.

**Figure 2:**
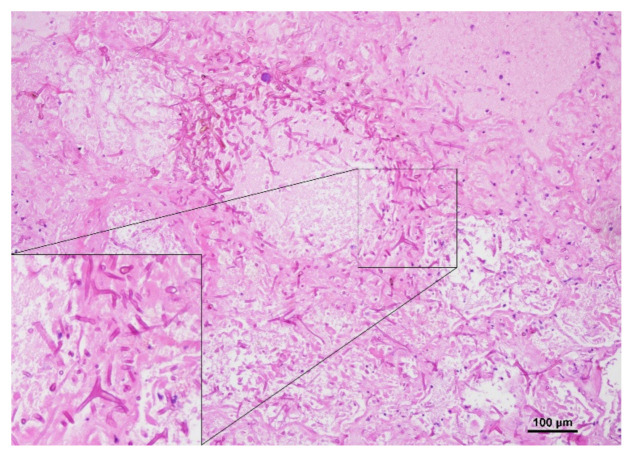
PAS (periodic acid–Schiff) staining of a postmortally acquired lung tissue specimen reveals fungal hyphae in necrotic lung tissue.

A full autopsy was performed, and microscopy of lung tissue showed tissue invasive nonpigmented fungal hyphae (Figure 2). To specify the fungal pathogen, internal transcribed spacer (ITS) sequencing from lung tissue was performed and revealed fungal DNA 100% homologous to *Rhizopus microsporus*. Thus, the patient was diagnosed postmortem with invasive pulmonary mucormycosis due to *Rhizopus microsporus* with no signs of dissemination. SARS-CoV-2 PCR from a throat swab performed postmortem was positive with a cycle threshold of 28, whereas PCR from lung tissue was negative.

## 3. Discussion

Here we report a case of a patient with AML and severe COVID-19 who was diagnosed postmortem with invasive pulmonary mucormycosis.

Invasive fungal infections (IFI) in critically ill COVID-19 patients are now a well-known threat to our patients. The vast majority of these patients are suffering from COVID-19 associated pulmonary aspergillosis (CAPA) [3]. Other IFIs, including invasive candidiasis or *Saccharomyces cerevisiae,* are also reported but CAPA is the dominating IFI in critically ill COVID-19 patients [4,5,6]. To date, only very few cases with mucormyosis are published [7,8] but none of them presented with pulmonary mucormycosis. The patient reported here was treated for secondary AML and developed severe COVID-19 during hospital stay, requiring ICU treatment. Due to severe COVID-19 ARDS the patient was treated with glucocorticoids and tocilizumab. Furthermore, intensive chemotherapy combined with remaining MDS resulted in a prolonged neutropenic phase. Thus, there were several risk factors for developing severe opportunistic infections such as pulmonary mold infections, and we primarily assume that the combination of these risk factors (underlying disease plus severe COVID-19 with ARDS plus corticosteroid treatment) triggered the emergence of mucormycosis in this patient. As all high-risk patients for IFIs at our center, the patient was closely screened with fungal biomarkers in serum samples (galactomannan, 1,3-ß-D-glucan) during ICU stay. Even though serum GM screening in most CAPA patients remains negative due to the predominantly airway invasive growth of *Aspergillus* [3], the patient reported here was neutropenic over a long period of time, increasing the sensitivity for blood biomarkers such as GM for CAPA. However, a routinely available fungal biomarker for mucormycosis is lacking, making it even more complicated to diagnose the disease accurately and rapidly.

Respiratory deterioration in a severely immunocompromised patient with COVID-19 should promptly trigger performance of a high-resolution CT scan and consecutive BAL. However, performing a CT scan in a mechanically ventilated and critically ill COVID-19 patient is logistically very challenging, whereas chest X-rays are usually available bedside and are therefore standard imaging procedure among COVID-19 patients on ICU. Regular bronchoscopies are also avoided and only performed if clinically indicated due to the risk of airborne transmission of SARS-CoV-2. These factors complicate the diagnosis of secondary pulmonary infections, including mucormycosis. Although BAL was performed in our patient at the time of deterioration, no *Mucorales* growth was detected in culture, underlying the limited sensitivity of BAL culture for mold detection. Consequently, voriconazole treatment was not replaced with other azoles with documented activity against Mucorales, and the diagnosis of mucormycosis was only established postmortem.

The report is intended to raise awareness for these devastating infections that may also complicate severe COVID-19; early appropriate and aggressive therapy is needed for a successful outcome of mucormycosis [9].
